# Exercise Training and Circulating Small Extracellular Vesicles: Appraisal of Methodological Approaches and Current Knowledge

**DOI:** 10.3389/fphys.2021.738333

**Published:** 2021-10-28

**Authors:** Ian A. J. Darragh, Lorraine O’Driscoll, Brendan Egan

**Affiliations:** ^1^School of Health and Human Performance, Dublin City University, Dublin, Ireland; ^2^School of Pharmacy and Pharmaceutical Sciences, Trinity College Dublin, Dublin, Ireland; ^3^Trinity Biomedical Sciences Institute, Trinity College Dublin, Dublin, Ireland; ^4^Trinity St. James’s Cancer Institute, Trinity College Dublin, Dublin, Ireland; ^5^National Institute for Cellular Biotechnology, Dublin City University, Dublin, Ireland; ^6^Florida Institute for Human and Machine Cognition, Pensacola, FL, United States

**Keywords:** endurance, exercise factors, exerkines, exosomes, myokines, skeletal muscle

## Abstract

In response to acute exercise, an array of metabolites, nucleic acids, and proteins are enriched in circulation. Collectively termed “exercise factors,” these molecules represent a topical area of research given their speculated contribution to both acute exercise metabolism and adaptation to exercise training. In addition to acute changes induced by exercise, the resting profile of circulating exercise factors may be altered by exercise training. Many exercise factors are speculated to be transported in circulation as the cargo of extracellular vesicles (EVs), and in particular, a sub-category termed “small EVs.” This review describes an overview of exercise factors, small EVs and the effects of exercise, but is specifically focused on a critical appraisal of methodological approaches and current knowledge in the context of changes in the resting profile small EVs induced by exercise training, and the potential bioactivities of preparations of these “exercise-trained” small EVs. Research to date can only be considered preliminary, with interpretation of many studies hindered by limited evidence for the rigorous identification of small EVs, and the conflation of acute and chronic responses to exercise due to sample timing in proximity to exercise. Further research that places a greater emphasis on the rigorous identification of small EVs, and interrogation of potential bioactivity is required to establish more detailed descriptions of the response of small EVs to exercise training, and consequent effects on exercise adaptation.

## Introduction

When individuals undertake repeated bouts of exercise (i.e., exercise training), acute molecular responses and chronic adaptive changes occur that result in functional changes at the levels of cells, tissues, organs, and systems. These changes ultimately produce improvements in health status, and/or exercise capacity and performance, amongst other phenotypic changes. Currently, the totality of processes that regulate exercise adaptation are incompletely understood, and uncovering novel mechanisms that contribute to, or are characteristic of, this process represents a central topic of research for exercise physiologists (Egan and Zierath, 2013). Acute exercise induces the enrichment in circulation of a vast array of factors including metabolites, several RNA species, and peptides/proteins (Contrepois et al., 2020). These may be derived from a variety of sources that include contracting skeletal muscle, among other prominent examples such as endothelial, cardiac, hepatic, and adipose tissues (Estébanez et al., 2020; Murphy et al., 2020). The physiological relevance of the enrichment of many of these factors is mostly unknown, but they may contribute to regulation of homeostasis and substrate metabolism during and after exercise (Murphy et al., 2020), and/or serve as the initiating signals for the adaptations that occur in response to repeated bouts of exercise (Hoffmann and Weigert, 2017). Under specific conditions, some of these factors have been classified with established labels, namely “myokine” for peptides and proteins released from skeletal muscle in response to exercise (Murphy et al., 2020), and “exerkine” for protein or RNA factors enriched in response to exercise, but with ambiguous tissue origin (Safdar et al., 2016). However, these terms are often used interchangeably, or in a manner that is misaligned to their definition (Eckel, 2019). Therefore, to avoid conflicting terminologies, within this article we operationally refer to all circulating molecules that are evidently responsive to exercise with the collective term of “exercise factors” (Goldstein, 1961; Severinsen and Pedersen, 2020). This term is generic and all-encompassing to relevant molecule species (i.e., metabolites, nucleic acids, and proteins) from any cellular source. We also consider a circulating exercise factor as any factor that is reported to demonstrate responsiveness to either acute exercise or chronic exercise training. This approach enables acknowledgment of a variety of potential responses across molecules and conditions, without having to provide excessive clarifications or redefinitions when divergences between individual molecules are present.

Extracellular vesicles (EVs) are a family of lipid bilayer encapsulated molecules that have regulated released from all nucleated cell types and are measurable in most common biofluids (e.g., blood, sweat, and urine; van Niel et al., 2018). EVs contain heterogenous bioactive molecules or “cargo” (e.g., metabolites, nucleic acids, and proteins), which are derived from their cell of origin, and are capable of being taken up into cells that are either proximal or distal to their site of release. These features have led to the assumption that the primary function of EVs is to serve as communicative “messages” between cells (Yáñez-Mó et al., 2015). Recently, it has been reported that EVs undergo circulating enrichment in response to acute exercise [reviewed by (Estébanez et al., 2020) and (Nederveen et al., 2021)] and this has produced speculation that EVs may represent a medium of transport for exercise factors (Safdar et al., 2016; Murphy et al., 2020). Under current models, the principal idea considered is that the mechanism by which many exercise factors appear in circulation is packaged as cargo within released EVs (Estébanez et al., 2020). This is suggested to serve as a means of protecting certain factor types (e.g., RNA) from degradation, while also explaining how proteins devoid of secretory peptide sequences may still enrich in circulation in response to exercise (Safdar et al., 2016). Interestingly, some subtypes of exercise factors have been reported to exhibit differential profiles between exercise-trained and sedentary individuals under resting conditions using metabolomic and proteomic analyses (Wei et al., 2018; Monnerat et al., 2020). Here we define “profiles” as the detection of individual factors as present or absent, and/or changes in their estimated abundance(s) in circulation. The observation of differential profiles suggests that exercise training may induce changes to the circulating milieu that are somewhat durable, rather than only transient and present during, and soon after, exercise.

Much of the current interest regarding the response of EVs to exercise has been focused on a subfraction of EVs in smaller size ranges (diameters of 50–150 nm), termed “small EVs” (Estébanez et al., 2020; Vechetti et al., 2020; Nederveen et al., 2021), with several studies having now investigated the effect of exercise training on the circulating profile (particle number, concentration/abundance, cargo, and/or cargo density) of small EVs (Chaturvedi et al., 2015; Bei et al., 2017; Bertoldi et al., 2018; Ma et al., 2018; Hou et al., 2019; Barcellos et al., 2020; Castaño et al., 2020; Nair et al., 2020; Xiang et al., 2020; Estébanez et al., 2021; Gao et al., 2021).

The primary aim of this review is to critically appraise whether changes in the profile of small EVs under resting conditions is expectable as a characteristic response to exercise *training*. To achieve this aim, we will outline the general paradigm of exercise factors and their response to acute exercise and chronic exercise training, in addition to discussion of small EVs in the same context, and what is currently known about the bioactivity of small EVs altered by exercise training. We will also highlight key methodological considerations for both the identification (separation and characterization) of small EVs, and the interpretation of exercise training studies given the critical importance of these aspects to our aim.

## Overview of the Response of Exercise Factors to Acute Exercise

For each subcategory of exercise factors (i.e., metabolites, nucleic acids, and proteins), the number of individual molecules that are reported to change in response to acute exercise is often estimated to be hundreds (Fernández-Sanjurjo et al., 2018; Sakaguchi et al., 2019; Guseh et al., 2020). A small number of molecules may decrease in abundance in response to acute exercise, but it is generally considered that the majority of changes in exercise factors are in the form of increased circulating abundance(s; Fernández-Sanjurjo et al., 2018; Son et al., 2018; Murphy et al., 2020; Severinsen and Pedersen, 2020). Some individual factors are well-described and understood in terms of the kinetics of their response to exercise and subsequent metabolic and/or molecular effects, such as the metabolite lactate (van Hall, 2010; Brooks et al., 2021), and the myokine interleukin-6 (IL-6; Pedersen and Febbraio, 2008). Mention of the latter molecule is of particular importance as an illustration of a prototypical exercise factor. During and soon after a bout of aerobic exercise, circulating IL-6 is robustly (and sometimes substantially) increased, is mostly derived from contracting skeletal muscle (Pedersen and Febbraio, 2008), and exerts relevant effects during (e.g., enhanced hepatic glucose output and adipose tissue lipolysis) and after (e.g., enhanced insulin sensitivity in skeletal muscle and enhancing pancreatic β-cell mass) exercise (Severinsen and Pedersen, 2020).

Collectively, these observations regarding IL-6 are seminal, as they established an intellectual foundation for the paradigm of how many exercise factors are now generally presumed to function. However, for the majority of exercise factors, limited information is available beyond (sometimes inconsistent) reports that indicate a change in circulating concentration(s) in response to acute exercise. The details regarding subcategories of exercise factors (e.g., potential bioactivities, variability within and between subcategories, and variability in response to different types of exercise) are beyond the scope of this review, but are discussed elsewhere in relation to metabolites (Sakaguchi et al., 2019; Kelly et al., 2020), RNAs (Sapp et al., 2017; Fernández-Sanjurjo et al., 2018), and proteins (Hoffmann and Weigert, 2017; Eckel, 2019; Severinsen and Pedersen, 2020). As EVs are proposed as potential carriers of many of these exercise factors (Safdar et al., 2016), there is an implication that the circulating response of EVs to acute exercise should mimic the general response of exercise factors. In the case of an exercise-induced “increase,” this change would manifest through a combination of an absolute increase in the circulating abundance of EVs, a change in cargo profile, and/or an increase in cargo density per EV.

## What Are Extracellular Vesicles?

Extracellular vesicle is a generic term used to describe any lipid bilayer-encapsulated particle that is naturally-released by cells and is incapable of independent replication (Théry et al., 2018). At rest, EVs are continually released and removed from circulation (Matsumoto et al., 2020), but can demonstrate enhanced release in response to physiological stimuli; for example, in response to hypoxia or shear stress in vascular endothelial cells (Hromada et al., 2017). Circulating EVs elicit bioactivity through uptake and delivery of their molecular cargo to recipient cells, often different from the tissue of origin, and are implicated in the regulation of physiological processes, such as coagulation and antigen presentation (Yáñez-Mó et al., 2015).

For exercise physiologists, interest in EVs developed greater traction with the observation that many circulating proteins as candidate exercise factors did not contain signal (secretory) peptide sequences, and yet were present in online EV expression databases (Safdar et al., 2016). Currently, rigorous investigations of EVs in the context of response to acute exercise are limited, but reports are often interpreted as indicating EVs respond in a manner that mirrors that of exercise factors more broadly. Specifically, that EVs are transiently-enriched in circulation during and after exercise, and elicit relevant bioactivity through the delivery of their cargo to recipient cells (Vechetti et al., 2020; Nederveen et al., 2021).

Extracellular vesicles are broadly separated into three subtypes, which are delineated by their mechanism of biogenesis, namely (i) exosomes, (ii) ectosomes (a.k.a. microparticles or microvesicles), and (iii) apoptotic bodies (van Niel et al., 2018). Exosomes represent released intraluminal vesicles (ILVs), which are derived from multi-vesicular bodies (MVBs) that originate with inward budding of the plasma membrane. Because of their mechanism of origin, exosomes are expected to fall within the size range of MVB-associated ILVs, i.e., ∼50–150 nm (van Niel et al., 2018; Kalluri and LeBleu, 2020). Ectosomes are defined as EVs which originate via direct shedding from the plasma membrane. The exact mechanism of ectosome shedding in non-apoptotic cells is less well-described, but is suggested to involve altered membrane asymmetry via cytoskeleton remodeling mediated by Ca^2+^-sensitive aminophospholipid translocases (“floppases” and “flippases”). Ectosomes have a broader reported size range of 50 nm to 1 μm (van Niel et al., 2018). Exosomes currently dominate the general interest in EVs in the domains of exercise physiology, metabolism and adaptation (Safdar and Tarnopolsky, 2018; Vechetti et al., 2020). As such, throughout this review, we will only consider reports which have endeavored to separate and characterize EVs that are within the size-range of exosomes (i.e., 50–150 nm). However, as EVs with similar physical characteristics to exosomes can bud directly from the plasma membrane (and therefore are not exosomes; van Niel et al., 2018) and technical challenges regarding the isolation of “pure” samples of individual EV subtypes (*vide infra*), hence we refer to these particles using the more collective term of “small EVs” (Théry et al., 2018).

## Separation and Characterization of Extracellular Vesicles

Pertinent to any discussion of EVs is acknowledgment that there are currently no routinely-used direct methods for the characterization or quantification of EVs from biofluid samples. Instead, separation of biofluid fractions and multiple methods for identification and characterization are required to identify both the presence and quantity of EVs in a sample. This has implications regarding the inferential value of individual experimental studies concerning small EVs in any context. To enable critical discussion of relevant exercise studies, a brief overview of how EVs are currently recommended to be identified and analyzed by the International Society of Extracellular Vesicles (ISEV) via the MISEV2018 Guidelines is provided subsequently and are summarized in Table 1. Readers are also referred to the comprehensive position stand which details these guidelines (Théry et al., 2018).

**TABLE 1:** Summary of the minimal information for the study of extracellular vesicles (MISEV) guidelines.

Small EV characteristic	MISEV recommendation	Approaches	Analytic considerations
Quantity of EVs in a sample	- A quantified estimate of both the source of EVs (e.g., extracted whole blood/plasma volumes) and EVs themselves should always be provided	- Particle quantification (e.g., NTA, flow cytometry) - Total protein quantification (e.g., SDS page) - Total lipid quantification (e.g., SDS page)	- None of these methods exclusively quantify EVs - EV quantification is improved when methods are used in conjunction and to provide ratios indicative of purity, e.g., protein/particle or lipid/particle
EV marker identification	- At least three positive protein markers associated with EVs; including at least one transmembrane protein and one cytosolic protein - A purity control such as proteins that are identified as common contaminants (e.g., lipoproteins in plasma)	Traditional methods of protein identification (e.g., Western blot)	- “Mixed” signal may be determined across positive markers and therefore it is important multiple markers are used - High contaminant signal may have implications for interpretation of some quantification methods (e.g., NTA)
Characterization of single vesicles	- At least two different, but complimentary, methods of vesicle visualization should ideally be employed	- Microscopic-based techniques (e.g., electron or atomic force microscopy) - Single particle analyzers (e.g., NTA)	- Most microscopy techniques are not interchangeable in terms of the information they provide - Different techniques may need to be employed depending on the EV size range of interest

*Modified from Théry et al. (2018).*

Most contemporary EV separation techniques involve separation of particles from a biofluid sample (e.g., blood, sweat, milk, and saliva) based on either physical properties (such as size and density), specific expression of EV marker proteins, or a combination of both [reviewed by (Doyle and Wang, 2019)]. Currently numerous methods are used to separate small EVs from biofluids, the most common of which are differential ultracentrifugation, density gradient separation, size exclusion chromatography, and immunoprecipitation (Cocozza et al., 2020).

While these common techniques all may be used individually to separate small EVs, some may also be employed in combination for additive effects (Théry et al., 2018). However, the parameters by which these techniques work to separate particles tend to overlap across EV subtypes and various other molecules, thus it is often considered unavoidable that preparations of EVs will contain a heterogenous mix of particles, e.g., several EV subtypes with similar physical or expression characteristics (Théry et al., 2018; Cocozza et al., 2020), and non-EV molecules such as argonaute proteins (Arroyo et al., 2011), lipoproteins (Jamaly et al., 2018), and exomeres. The latter are secreted, bioactive and non-membranous protein complexes with diameters of ∼35 nm (Zhang et al., 2018, 2019). Accordingly, ISEV recommends referring to EVs within separated samples using clearly-defined operational terms based on identified physical and/or biochemical characteristics of particles within said samples. For example, instead of referring to EVs by the labels of specific subtypes, isolated EVs should be classified as “small” or “medium/large,” and fixed size ranges for each term be declared, which can be determined at researchers’ discretion. Alternatively, EVs can be termed based on the positive detection for specific markers (e.g., CD63^+^ EVs), or the use of multiple defined parameters together such as “small CD63^+^ EVs” (Théry et al., 2018).

Once small EVs have been separated from biofluid, it is then essential to validate the presence of small EVs and provide an estimate of small EV quantity, particularly in circumstances where enrichment is presumed to occur (e.g., exercise; Safdar et al., 2016; Vechetti et al., 2020). Broadly, the MISEV2018 guidelines recommend use of three principal indicators to identify the extent of small EV presence in a biofluid sample (Table 1). These are the quantification of particles in a small EV size range; the detection of marker proteins associated with small EV membranes and contaminants; and the visualization of small EVs derived from a sample (Théry et al., 2018).

The first indicator is the quantification of small EVs involving the estimation of the size range and concentration of particles within the sample. This enables determination of the distribution of particle diameters contained within a sample, and subsequent estimation of the concentration of particles that fall within the size range of small EVs (50–150 nm). Several methods are available for determining physical characteristics of these particles, but the most common technique employed in exercise studies is nano-particle tracking analysis (NTA). This technique measures the Brownian motion of individual particles via detection of scattered light, and by employing the Stokes-Einstein equation to determine diffusion coefficients can estimate distributions of particle concentration and diameter (Doyle and Wang, 2019). Traditional or modified methods of flow cytometry are also sometimes employed for the quantification of EVs by particle count (Welsh et al., 2020). Additionally, estimating total quantity of protein or lipid is useful as a global indication for determining the relative abundance of specific small EV markers or common contaminants (e.g., lipoproteins). These are recommended to be estimated via standard approaches, e.g., BCA assay or global protein stain of SDS-PAGE, and can also be usefully combined to estimate sample characteristics like unit protein per particle (Théry et al., 2018). The second indicator required for small EV enrichment is the characterization of common markers of EV status, which are generally “validated” small EV surface marker proteins such as CD63 (van Niel et al., 2018). Identification and quantification of at least three markers is recommended, encompassing an EV-associated protein and/or cytosolic protein, and at least one negative protein marker. The third, and final, indicator is the visual characterization of single vesicles, which is most commonly accomplished by using transmission or scanning electron microscopy, but may also employ techniques that can visualize vesicle topography, such as atomic force microscopy (Théry et al., 2018; Doyle and Wang, 2019).

Importantly, when considering the presence or bioactive effects of small EV cargoes, endeavoring to achieve a reliable indication of high abundance of small EVs in the sample is important, because some non-EV molecules that co-exist in separated samples may also serve as carriers for factors that are proposed as bioactive cargo of small EVs, such as microRNA (miRNA) and protein (Arroyo et al., 2011; Zhang et al., 2019). Subsequently, to determine any roles or responses of small EVs to acute exercise or exercise training, it is essential that a rigorous characterization of small EVs is made. The technical approach (e.g., the number and types of methods) required to characterize the presence of EVs in a sample is extensive, and may be challenging to accomplish. However, when considering the question of whether small EVs represent an important “carrier” of exercise factors, the extent to which any given report demonstrates the presence/quantity of small EVs will have substantial influence on the inferential value of the reported results.

## Changes in the Small Extracellular Vesicle Profile in Response to Acute Exercise

**FIGURE 1:**
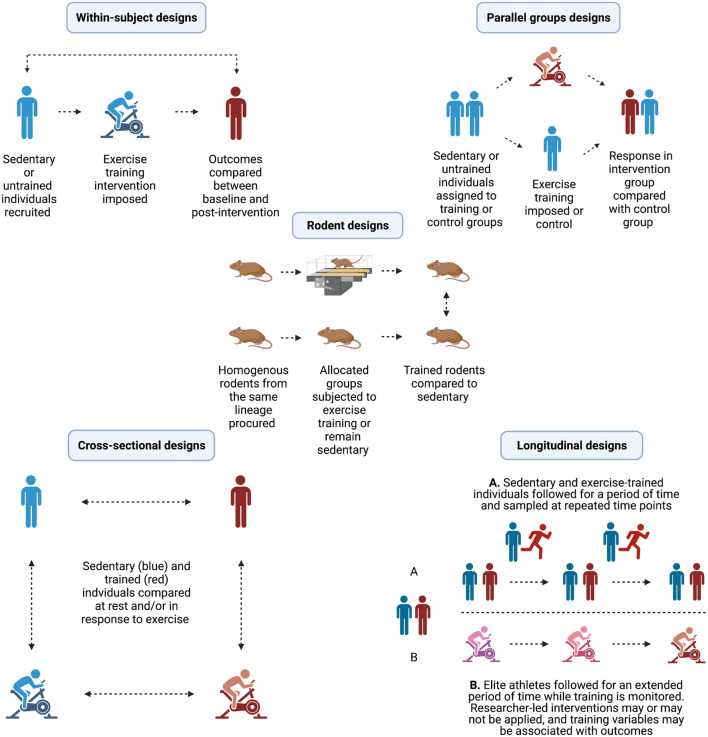
Overview of study designs used to investigate the effect of exercise training on physiological and/or performance outcomes.

When investigating the response to acute exercise, the profile of small EVs would then ideally be compared between pre-, during and/or post-exercise samples using a multi-methods approach based on ISEV guidelines (Figure 1). Namely, enrichment would be evident as greater particle concentration (e.g., a greater NTA or flow cytometry signal), and quantities of total protein and small EV protein markers would be measurable in post-compared to pre-treatment (e.g., exercise) samples, together with a consistent (and ideally low) indication of contamination within preparations of small EVs from both pre- and post-treatment (Théry et al., 2018). The confidence with which enrichment could be inferred would then be based not on the signal of a single indicator, but instead on the general signal across multiple independent methods (Figure 1).

Currently several studies have investigated elements of the response of circulating small EVs to acute aerobic (Frühbeis et al., 2015; Guescini et al., 2015; Bertoldi et al., 2018; D’Souza et al., 2018; Lovett et al., 2018; Oliveira et al., 2018; Whitham et al., 2018; Brahmer et al., 2019; Yin et al., 2019; Karvinen et al., 2020; Rigamonti et al., 2020; Gao et al., 2021; Neuberger et al., 2021; Zhang et al., 2021), resistance (Annibalini et al., 2019; Just et al., 2020; Vechetti et al., 2021) or combined plyometric and downhill running (Lovett et al., 2018) exercise. Most of these studies have attempted to characterize circulating small EVs separated from plasma (Frühbeis et al., 2015; Guescini et al., 2015; D’Souza et al., 2018; Lovett et al., 2018; Whitham et al., 2018; Annibalini et al., 2019; Brahmer et al., 2019; Yin et al., 2019; Just et al., 2020; Rigamonti et al., 2020; Gao et al., 2021; Neuberger et al., 2021; Vechetti et al., 2021), although four studies have separated small EVs from serum (Bertoldi et al., 2018; Oliveira et al., 2018; Karvinen et al., 2020; Zhang et al., 2021).

An important point is that platelets are purported to be the largest contributors to the circulating EV pool (Yáñez-Mó et al., 2015). Therefore, results between studies that separate EVs from serum and plasma, which are rich in and depleted of platelets, respectively, will have inherit differences in their EV profiles, particularly as platelets may contribute to the exercise-associated small EV pool (Brahmer et al., 2019). This contribution is likely to be more apparent in preparations of small EVs separated from serum, and may influence differences in indications of small EV responses to exercise (e.g., enrichment) between studies.

In addition to differences in biofluid sources, these studies have not had consistent methodological approaches, which hampers broad conclusions on the nature of the response of circulating small EVs to acute exercise. For example, four studies have only examined changes in the concentration of specific miRNAs from plasma samples taken before and after aerobic exercise (Guescini et al., 2015; D’Souza et al., 2018; Yin et al., 2019; Karvinen et al., 2020). The inference was that these miRNAs represent small EV cargo. However, in each of these studies, miRNA concentrations (transcript abundance) were quantified in both pre- and post-exercise samples, but additional methods of small EV characterization were only applied to post-exercise samples (Guescini et al., 2015; D’Souza et al., 2018; Karvinen et al., 2020) or not at all (Yin et al., 2019). Problematically, this type of technical approach provides no indication as to whether the abundance of small EVs was in fact changed by the exercise bout and cannot associate changes in the concentration of these miRNAs with a concomitant change in the estimated abundance of small EVs. Given the heterogenous nature of preparations of small EVs, it is difficult to conclude that the data from these studies are definitively indicative of a change in small EV profile in response to acute exercise.

Ten studies have employed a single methods approach for determining small EV enrichment using either NTA (Lovett et al., 2018; Annibalini et al., 2019; Just et al., 2020; Rigamonti et al., 2020; Vechetti et al., 2021), flow cytometry (Bei et al., 2017; Gao et al., 2021), or Western blot (Bertoldi et al., 2018; Neuberger et al., 2021; Zhang et al., 2021) to estimate small EV enrichment in response to acute aerobic (Bei et al., 2017; Bertoldi et al., 2018; Rigamonti et al., 2020; Gao et al., 2021; Neuberger et al., 2021), resistance (Annibalini et al., 2019; Just et al., 2020; Vechetti et al., 2021), or combined plyometric and downhill running (Lovett et al., 2018) exercise. Each of these studies, except one (Lovett et al., 2018), observed an increase in their respective measures in within preparations of small EVs taken after exercise, which potentially indicates an exercise-induced enrichment in small EVs. However, using only measures of particle count is problematic because these only provide an indication of the concentrations of particles with specific size characteristics, which in practice would have low specificity for individual subtypes of EVs. For example, the use of NTA alone can reduce the accuracy of results as this measure is highly sensitive to factors such as nutrition status (Mørk et al., 2016; Jamaly et al., 2018; Brahmer et al., 2019).

Four studies have employed multi-method approaches in response to acute aerobic exercise (Frühbeis et al., 2015; Oliveira et al., 2018; Whitham et al., 2018; Brahmer et al., 2019). Two of these studies have seen increases in both NTA signal and protein concentration within preparations of small EVs derived from human plasma (Frühbeis et al., 2015; Whitham et al., 2018). One study employing a proteomics approach observed an exercise-induced increase in the abundance of 299 proteins (some of which were markers of small EVs such as CD81), which coincided with an increase in NTA signal within preparations of small EVs (Whitham et al., 2018). Another study estimated an increase in the small EV marker proteins FLOT1 and HSP70 via Western blot, which also coincided with an increase in NTA signal comparing pre- and post-exercise samples (Frühbeis et al., 2015).

Small EV preparations from Wistar rats exposed to acute low, moderate or high intensity exercise demonstrated greater concentration of small EV-sized particles, total protein and CD63 abundance compared to samples derived from a sedentary control group. Importantly, APOIV, a lipoprotein and indicator of contamination preparations of separated EVs, was present but had similar abundance across all conditions (Oliveira et al., 2018). Another study using a pre-post analysis of acute exercise in humans with minimal lipoprotein contamination used NTA and Western blot analysis of small EV markers (i.e., CD9, CD63, Syntenin, CD41b, TSG101, and CD81), or a novel multiplex array that enables concurrent detection of 41 surface EV proteins (Brahmer et al., 2019). These methods produced mixed results by observing no differences in small EV-sized particle concentration by NTA, but increases in multiple markers of small EVs via Western blot and multiplex assay approaches (Brahmer et al., 2019).

In this section, we have focused on appraising the approaches employed for determining whether small EV enrichment occurs, rather than focusing on the specifics of the exercise bouts, sample timing, or the specific changes in cargo or concentration during and after exercise. These specific details have been the subject of several recent reviews (Estébanez et al., 2020; Vechetti et al., 2020; Nederveen et al., 2021), and it is now often accepted that small EVs are enriched in circulation in response to acute exercise (Safdar et al., 2016; Whitham and Febbraio, 2016; Murphy et al., 2020; Vechetti et al., 2020), despite the methodological limitations of studies to date. Nevertheless, rigorously measuring small EV enrichment in response to exercise remains challenging to accomplish, as multi-method approaches are required and agreement across selected methods may not always be apparent (Brahmer et al., 2019). However, the majority of studies to date have not provided sufficient characterization of small EVs to definitively conclude that enrichment has occurred. That said, of the limited number of studies where multi-method approaches have been applied, acute exercise arguably does induce an enrichment of small EVs in circulation. This is consistent with the general response of exercise factors, and therefore the proposed role of small EVs as a medium through which exercise factors are transported in circulation.

## Study Design Considerations for Investigating the Adaptive Response to Exercise Training

Prior to considering whether exercise factors and/or small EVs are responsive to exercise training, it is salient to consider the various study design approaches that are employed to study exercise adaptation (Figure 1). Describing the features, advantages and disadvantages of each of these designs is pertinent to the primary aim of this review, as acknowledgment of the limitations of study designs also informs the extent to which inferences can be made regarding measured changes in small EV profiles associated with exercise training. There are three broad study designs that can be employed to examine adaptive changes in response to exercise training, namely pre-post intervention studies, cross-sectional studies, and longitudinal/prospective cohort studies.

### Pre-post Intervention Study Designs

Pre-post intervention study designs represent a direct approach for investigating change in physiological and performance phenotypes in response to exercise training. There are numerous ways that these designs can be implemented including approaches of randomized or non-randomized control trials of parallel groups, single group designs, and detailed *n* = 1 case studies, amongst others as detailed elsewhere (Hecksteden et al., 2018). However, the common feature within this design category is the exposure of participants to a period of structured exercise training focused on changing some aspect of health or fitness, and a subsequent comparison of physiological and/or performance outcomes. In human trials, this outcome is achieved generally by either a within-group pre-post comparison (single group design), or between-group comparison to a sedentary control group (parallel group design).

There are also well-established models of exercise training in rodents, which provide the advantage of a high degree of control over homogenous groups, and arguably can provide more detailed mechanistic insight into adaptations to exercise. The advantages provided by control over ambulatory activity, feeding times and dietary composition, environmental conditions, sleep/wake cycles, and compliance with training often not possible in human studies. However, these experimental advantages must be tempered with the caution that innate differences (e.g., morphological and metabolic) between rodents and humans can make it difficult to reproduce some findings between species (Fuller and Thyfault, 2021). Additionally, within-subject designs in rodents are often impossible to conduct for invasive measures such as muscle and blood sampling. This is principally due to methodological limitations such as requiring the excision of whole skeletal muscles, or physiological limitations such as blood volumes required for downstream analyses. For example, rats have a total blood volume of ∼10 to 25 mL on average depending on bodyweight (Lee and Blaufox, 1985), whereas mice have generally less than 2 mL (Riches et al., 1973). Considering the volume of plasma required for many assays, including for the analysis of small EVs, where multiple independent assays are required and thus large blood volumes are desirable (Théry et al., 2018), sampling at serial timepoints in an individual rodent is unfeasible.

Therefore, exercise training studies in rodents typically involve randomized groups selected from a homogenous inbred lineage either subjected to a period of forced daily exercise training (e.g., treadmill running or swim training), or allocated as a group of sedentary controls. Groups are subsequently compared after being euthanized at the same time-point corresponding to the end of exercise training intervention. An important point to note is that with such a design, any differences observed between groups are technically cross-sectional in nature, i.e., differences between trained and untrained groups (see section “Cross-sectional studies”). Therefore, results should be described as characteristic of the trained state, rather than as training-induced changes in a phenotype or outcome variable.

In general, the primary advantage of training intervention studies in rodents or humans is that phenotypic changes can be attributed to certain characteristics of the exercise training stimulus controlled as independent variables such as the frequency, intensity, duration and type of exercise bouts. However, these interventions also suffer from notable limitations. For example, most training studies employ sedentary participants and relatively short intervention durations (e.g., several weeks to months; Hecksteden et al., 2018). However, even short (e.g., 2 weeks) interventions can result in substantial increases in aerobic fitness, and induce marked changes in transcripts and proteins in skeletal muscle (Perry et al., 2010; Egan et al., 2013). Sedentary individuals are the most responsive to the onset of an exercise training intervention, and therefore the changes that often occur in short interventions are unlikely to be reflective of what would continue to occur were the training intervention planned appropriately and continued in an extended fashion. Short-term training studies cannot be assumed to represent the same adaptive processes present in individuals who have extensive exercise training histories (e.g., years/decades), which are typically assessed in cross-sectional study designs (section “Cross-Sectional Studies”). Nor can the time course for the changes in physiological responses and/or performance outcomes that are observed in short-term training studies being interpreted as continuing in a linear manner.

A final consideration is that especially in studies of the molecular regulation of adaptation in humans, control groups are often absent and single group within-subject designs are employed. This type of design has implications regarding the extent that pre- to post-intervention differences can be accurately quantified because the absence of a control group does not allow analyses to account for factors such as the regression to the mean artifact, and/or random variability, ever-present and uncontrollable, in biological measurements between- and within-individuals (Atkinson and Batterham, 2015).

### Cross-Sectional Studies

Cross-sectional studies encompass a form of observational research that broadly involves an isolated comparison of measurements representative of traits of interest between members of distinct population groups (Levin, 2006). For effects of exercise training, cross-sectional studies tend to involve recruiting and stratifying participants into exercise-trained and sedentary or untrained groups, based on standardized criteria of fitness (e.g., maximal oxygen uptake, VO_2__max_; maximal power output, W_max_; one repetition maximum, 1RM), training history or competitive status (e.g., elite cyclist or powerlifter), and/or exercise performance (e.g., personal best running a set distance, or weight lifted in competition). Subsequently, differences in relevant resting measurements and/or physiological responses and performance outcomes are compared between groups. In these reports, group differences are then often inferred as the indications of the consequences of exercise training, with the reasoning that when other major confounders (e.g., age, sex, indices of health status) are controlled for, the largest determinant of difference between groups is the regular participation in specific type(s) of exercise training.

The advantage of this type of study design is that it is often less expensive, less of time burden, and relatively simple to perform in comparison to training interventions. This generally allows the collection of larger sample sizes at lower cost, and sometimes the recruitment of a higher caliber of trained participant such as elite athletes. The more likely participation of elite athletes in cross-sectional studies is due to having lower time commitments and generally not interfering with athletes’ training regimes. However, cross-sectional studies are limited mostly by the fact they only provide a “snapshot” at a specific moment in time, and therefore indicate the prevalence of traits or responses between exercise-trained or untrained groups at the time of measurement (Sedgwick, 2014). These studies are not capable of providing information on *how* a parameter of interest has changed over time to eventually qualify a participant for inclusion in the trained group. Additionally, some performance-associated traits such as VO_2__max_ may be relatively-stable (Edgett et al., 2018), and thus can provide some reliable ecological insight from a single timepoint measure.

This case is potentially less for many discrete biological measurements (such as circulating factors), which may have an inherent within-individual biological variability, often day-to-day and that is often unknown, or unaccounted for. For example, both of the exercise factors IL-6 and Fibroblast growth factor 21 (FGF21) are reported to have both innate diurnal and inter-individual variations in resting circulating concentrations (Sothern et al., 1995; Yu et al., 2011). Under these designs, the standardization of preparation for participants is paramount including, but not limited to, preceding days’ dietary intake, preceding night’s sleep, duration of fasting and morning ambulation for morning fasted samples, and time since last bout of exercise. Therefore, in cross-sectional studies where repeated experimental measures are not employed (e.g., duplicate or triplicate sampling of participants under similar resting or experimental conditions across several laboratory visits), the results of some measures may be less reliable, which could reduce inferential utility.

While in many cases between-group differences may most obviously be the consequence of differences produced by prolonged exercise training, quantifying the exact contribution of training to individual phenotypes is not possible in cross-sectional studies. Importantly, there is often no consensus on minimum performance or physiological thresholds that can be used to delineate an individual as “exercise-trained.” For example, one excellent approach is the physiology of road cyclists, wherein the categories of trained, well-trained, elite, and world class have been delineated based on training status, estimated by the weekly frequency of training sessions, the total volume of weekly training and the total year spent training; racing status estimated by the number of competition days per year and international ranking of the rider; and physiological capacity, estimated by common physiological parameters associated with performance (e.g., W_max_ and VO_2__max_; Jeukendrup et al., 2000). However, this approach is a rare exception as many studies opt to use different and sometimes arbitrary minimum thresholds for their trained participants. This lack of consistency can have the effects of making comparisons between studies more difficult and making the trained groups somewhat heterogenous and not divergent enough from the untrained group in order reveal meaningful between-group differences. For example, if a study categorizes a participant as trained if they demonstrate a VO_2__max_ > 60 mL kg^–1^ min^–1^, this approach may subsequently produce a group with individuals with VO_2__max_ values ranging 60–80 mL kg^–1^ min^–1^. This group would represent a mixed population group of trained, well-trained and elite athletes of similar physiological phenotypes, but in fact, they would likely be heterogenous on parameters such as training volume, lactate threshold, and mechanical efficiency. Indeed, obvious physiological differences between individuals within this example range have been reported, e.g., in citrate synthase activity, an indication of mitochondrial mass (Jacobs and Lundby, 2012). Lastly, cross-sectional studies often request that participants do not engage in moderate-to-vigorous exercise for a fixed period of time (e.g., ∼24 to 48 h) before coming to the laboratory, but it is often difficult to validate adherence to this recommendation, with the problem being that the residual effects of acute exercise may also be present in these samples, i.e., the “last bout effect,” which describes residual, but transient, physiological changes induced by acute exercise that extend beyond the cessation of exercise (e.g., for 24–48 h), but do not manifest as an adaptive response to long-term exercise training (see section “‘Last Bout Effect’: The Importance of Sample Timing”).

### Longitudinal and Prospective Cohort Study Designs

Like cross-sectional designs, longitudinal study designs are a form of observational research, with the principal difference being that participants are monitored over an extended period of time, and re-sampled at various intervals (Caruana et al., 2015). Some studies may also employ overlapping longitudinal and interventional designs, although these reports tend to be rare in exercise science (Hecksteden et al., 2018).

Longitudinal studies possess the principal advantages of cross-sectional studies (i.e., easier to measure larger sample sizes compared to a structured/supervised training intervention) and some of the limitations (heterogenous groups of participants, limited standardization of participant preparation prior to measurements, arbitrary thresholds of group qualification). However, this design may provide additional insight into the reliability of measures and the stability of traits within- and between-groups. Such between-group comparisons would require recruitment of a control or comparator group with the design then being a prospective cohort study.

Longitudinal and prospective cohort designs are often also applied to measure the physiological and performance development of well-trained and elite individual athletes and teams preparing for competitive events (Jones, 1998; Gabbett, 2005; García-Pallarés et al., 2010). While the latter is not specifically a training intervention *per se* (as the researchers often have no direct control over the training of the participants), these types of observational design does provide insight into long-term development that can occur in conjunction with regular, intensive exercise training. However, these studies can be confounded by concurrent interventions employed by athletes (e.g., nutrition and recovery strategies), and fluctuations in training strategies across monthly or annual cycles that may not necessarily be tracked extensively by researchers. These confounders can make it more challenging to associate which specific elements of the exercise training process have the largest proportional influences on specific physiological or performance outcomes.

### ”Last Bout Effect”: The Importance of Sample Timing

An important consideration across all types of study purporting to measure adaptive changes with exercise training is the proximity of measurement of the outcome interest to the final exercise bout of the training intervention, or most recent exercise bout in the case of cross-sectional designs. This consideration is especially important for outcomes with short half-lives of response/decay, such as changes in circulating parameters, but less important for outcomes with longer half-lives of response/decay. An example of the latter is that one effect of prolonged resistance exercise training is an increase in muscle mass (Egan and Zierath, 2013), which can be said to be a chronic adaptation given that it persists for several days and weeks after training cessation before declining steadily over several months (Mujika and Padilla, 2000a, b).

In contrast, exercise elicits obvious beneficial effects as improvements in glycaemic control and insulin sensitivity, but these are likely transient and related to the most recent bout of exercise. For example, a single bout of exercise modestly lowers blood glucose concentrations in the immediate post-exercise period in patients with type 2 diabetes (Minuk et al., 1981), but also improves whole-body insulin sensitivity for up to 48 h after exercise cessation (Mikines et al., 1988; Perseghin et al., 1996; Koopman et al., 2005). The additive effect of repeated bouts of exercise, i.e., training, over and above those that are seen in the aftermath of an acute bout of exercise in isolation is relatively modest (Perseghin et al., 1996). In cross-sectional terms, although well-trained athletes have markedly-enhanced insulin sensitivity compared to sedentary individuals (King et al., 1987), the habitual state of an athlete is between two individual exercise training bouts, thereby making it difficult to distinguish differences between acute responses and chronic effects of exercise. However, when well-trained or physically-active individuals cease training or reduce their daily activity, a decline in insulin sensitivity rapidly occurs toward sedentary levels at a time when declines in aerobic fitness or increases in adiposity are negligible (Heath et al., 1983; Krogh-Madsen et al., 2010). In turn, a single bout of exercise is sufficient to restore insulin sensitivity in these detrained individuals to their previously trained values (Heath et al., 1983), suggesting the effects of exercise for improved whole-body insulin sensitivity are mediated in large part by the acute effects of a single bout of exercise, rather than a generalized training effect, i.e., the absence of a synergistic effect.

Therefore, the physiological responses to a single bout of exercise, i.e., acute responses to exercise, can extend to 24 to 48 h after the cessation of an individual exercise bout, and thus some convergence exists between the effects of an acute bout of exercise and those effects associated with adaptation to exercise training. This phenomenon was coined by William L. Haskell (Haskell, 1994) as the “last bout effect” and proposes that some metabolic effects and purported health benefits of exercise (e.g., lowering of blood pressure or circulating lipoprotein profile) are attributable to the biological consequences of the most recent bout of acute exercise, rather than to a true training adaptation.

The overall implication is that in studies where the post-intervention, or cross-sectional, blood samples are taken within close proximity (e.g., <48 h) to the final, or most recent, training bout, respectively, it may be difficult to discriminate whether some outcomes represent a chronic adaptation to training, or an extended residual effect of the last exercise bout. This point is particularly salient when interpreting current literature on the effect of exercise training on the resting profile of circulating small EVs given the research designs employed to date as discussed in section “Does Exercise Training Alter the Resting Profile of Circulating Small Extracellular Vesicles?”

## Does Exercise Training Alter the Resting Profile of Circulating Exercise Factors?

A pertinent question to our understanding of the biological importance and mechanistic consequence of exercise factors is whether these factors function as transient, beneficial responses exclusively related to the enrichment of factors induced by acute exercise, or whether exercise training induces more persistent changes to circulating exercise factors that are measurable distally from acute exercise. Clearly, exercise training alters the *resting* proteome (Holloway et al., 2009; Ferreira et al., 2014; Padrão et al., 2016), transcriptome (Pillon et al., 2019), and metabolome (Castro et al., 2019; Klein et al., 2020) of tissues such as skeletal (Holloway et al., 2009; Padrão et al., 2016; Pillon et al., 2019; Klein et al., 2020) and cardiac (Ferreira et al., 2014) muscle. Assuming that the internal biomolecular composition of a cell influences the host of factors it releases into circulation (Uhlén et al., 2019), exercise training-induced changes in the resting profile of exercise factors is a physiologically-plausible outcome. Conversely, to reiterate the example of IL-6, in healthy individuals exercise training does not appear to alter resting plasma concentrations of IL-6, nor the kinetics of an exercise induced circulating response (Fischer et al., 2004). There are numerous other experimental studies that have investigated the influence of exercise training on the resting abundance or concentration of a small number of selected candidate miRNA and protein targets in circulation. Detailed discussion of these reports is beyond the scope of this review, but collectively the training-induced response is equivocal; some factors increase after a period of exercise training, while others decrease, with occasional inconsistencies in individual factors across studies. Again, variations in experimental design and sample timing described in the section “Study Design Considerations for Investigating the Adaptive Response to Exercise Training” must be considered, and interested readers are referred to reviews with appropriately collated tables of these studies for miRNA (Fernández-Sanjurjo et al., 2018) and protein (Son et al., 2018).

An alternative approach to measurement of hypothesis-driven candidate targets is unbiased, hypothesis-free omics approaches surveying the broadest possible profile of exercise factors, albeit exercise training studies paired with these approaches are limited to date. One example is in the response to a 4 month treadmill training intervention (20 min at 12–15 m/s per day, 6 day/week), which produced numerous differences at 24 h post-intervention in the resting plasma proteome of male Sprague-Dawley rats (54 proteins in higher abundance, 47 proteins in lower abundance) in trained compared to sedentary controls (Wei et al., 2018). Similarly, cross-sectional studies have reported differences in the resting plasma proteomes of moderately-trained male and female endurance athletes (16 proteins in higher abundance, 23 proteins in lower abundance) compared to sedentary controls (Santos-Parker et al., 2018), and resting plasma metabolomes of elite (6 metabolites in higher abundance, 9 metabolites in lower in abundance) compared to less well-trained endurance athletes (Monnerat et al., 2020). Therefore, there is some indication that exercise training does result in alterations to resting concentrations of exercise factors in circulation. Given the proposed role of small EVs as carriers of these factors we will subsequently appraise the effect of exercise training on the resting profile of circulating small EVs in the next section.

## Does Exercise Training Alter the Resting Profile of Circulating Small Extracellular Vesicles?

There are currently thirteen available reports (across twelve experimental studies) that have investigated the influence of exercise training on indicators of small EV abundance and/or cargo (summarized in Table 2). Under the assumption that small EVs are carriers of exercise factors and that exercise factors experience training-induced alterations in resting concentrations in circulation, there are several scenarios wherein *increases* could be represented. The first is alterations in the cargoes associated with preparations of small EVs, i.e., the same approximate quantity of circulating small EVs, but with individual molecules presenting as novel cargo, or as cargo of greater abundance, in exercise-trained states. In other words, an increase in the “density” of a specific cargo per EV, or novel molecules appearing in detectable abundances in circulating small EVs as a result of exercise training. The second is an increase in the quantity of circulating small EVs, i.e., a greater total abundance of cargoes in circulation, which is caused by an increase in the total quantity of circulating small EVs in exercise-trained states (i.e., an exercise training-induced increase in the concentration of circulating small EVs at rest). The third scenario would be some combination of these two scenarios (e.g., a greater quantity of both small EV concentration and cargo abundance). The next subsections will describe the relevant studies in both rodents and humans and whether any of these scenarios are observed.

**TABLE 2:** Studies in rodents or humans that have investigated the effect of exercise training on the profile of circulating small EVs either by examining the response to exercise training interventions or by performing cross-sectional comparisons of trained and untrained individuals.

Article	Study design and intervention	Sample time point after last exercise bout	Separation and characterization of small EVs	Abundance of small EVs	Changes in small EV cargo
			**Training intervention studies**		

Ma et al., 2018	- 8–10 week old male C57BL/6 mice - 4 weeks of Low or Moderate treadmill running compared to Sedentary controls (*n* = 4–6 per group) - Training: 60 min of either Low (5 m/min) or Moderate (10 m/min) intensity; 5 day/week	24 h	- Separation by differential ultracentrifugation, and magnetic microbead sorting of EPC-derived EVs by CD34/VEGFR2 - Particle size and concentration quantified by NTA - Western blot of positive EV markers CD63 and TSG101 and EPC markers CD34 and VEGFR2	- CD34^+^/VEGFR2^+^ EV particles greater in Low (↑1.9x) and Moderate (↑4.3x) compared to Sedentary and greater (↑∼2.3x) in Moderate compared to Low	- miR-126 measured by qPCR - ↑1.5x in Low vs. Sedentary - ↑2.3x in Moderate vs. Sedentary - ↑1.5x Moderate vs. Low
Hou et al., 2019	- 6 week old male Sprague-Dawley rats - 4 weeks of swim training intervention compared to Sedentary controls - Training: 1 week of progressively increasing duration, then 3 weeks of 2 × 90 min; 7 day/week - Minimum of *n* = 6 per assay, but total sample size not reported	24 h	- Separation by differential ultracentrifugation - Particle size and concentration quantified by NTA - Western blot of positive EV markers CD81 and Tsg101 - Small EVs visualized with TEM	- No differences between groups	- 765 miRNAs assayed (Illumina HiSeq 2500) with 14 miRNAs differentially expressed (> ± 2x and *P* < 0.05) - 12 miRNA confirmed by qPCR as differentially expressed in Trained vs. Sedentary - Increased: miR-3571, miR-1-3p, miR-342-5p, miR-122-5p, miR-196b-5p, miR-486, miR-208a-3p, miR-3591, miR-184, miR-760-3p, miR-99a-5p (all ↑∼1.8-2.6x) - Decreased: miR-191a-5p (↓∼60%)
Castaño et al., 2020	- 15 week old male C57BL/6 mice - 5 week treadmill running intervention compared to sedentary controls - Training: 15 × 2 min at 80% of maximal running speed with 2 min rest between efforts; speed increased by 2 m/min each week; day/week not reported - *n* = 5/6 per group	48 h	- Separation by differential ultracentrifugation - Particle size and concentration quantified by NTA, and abundance by acetylcholinesterase activity - Western blot of positive EV markers CD63, CD9 and HSP70 - Small EVs visualized with TEM	- No subjective differences in CD63, CD9 and HSP70 between groups - No differences in acetylcholinesterase activity in equal volumes of plasma between groups	- 378 miRNAs assayed by qPCR with 7 miRNAs differentially expressed in Trained vs. Sedentary - Increased: miR-133b-3p (↑11.0x), miR-205-5p (↑10.3x), miR-206-3p (↑9.6x), miR-133a-3p (↑9.5x), miR-19b-3p (↑2.7x), miR-30d-5p (↑2.2x) - Decreased: let-7g-5p (↓7.3x)
Estébanez et al., 2021	- Humans aged 70–85 year - 8 weeks of whole-body progressive resistance training (*n* = 28 M/F, 15/13) compared to Sedentary controls (*n* = 10; M/F, 1/9) - Training: 3 sets of 12–8–12 repetitions of 8 exercises of; 2 day/week	5 to 6 days	- Separation by differential ultracentrifugation - Western blot of positive EV markers CD9, CD14, CD63, CD81, Flot-1, and VDAC1	- Increase in CD63 was lower in Trained (6.8%) vs. Sedentary (42.5%)	- No difference between groups in miR-146-5p measured by qPCR
Garai et al., 2021	- Human males aged 23 ± 2 year (*n* = 14) - Concurrent aerobic and resistance exercise training three times per week for 6 months	At least 24 h	- Separation from pooled serum samples using Total Exosome Isolation reagent and traditional centrifugation - Particle size and concentration quantified by NTA, using unlabeled and labeled (CD63, CD81) preparations - Small EVs visualized with TEM	- No differences between groups	- 54 miRNA identified via NanoString multiplex array with 7 miRNAs differentially expressed in Post vs. Pre (FDR < 0.05) - Decreased: miR-21-5p (FC 0.73), miR-451a (FC 0.59), miR-130a-3p (FC 0.36), miR-15b-5p (FC 0.39), miR-199a/b-3p (FC 0.40), miR-223-3p (FC 0.45), miR-23a-3p (FC 0.49)

			**Cross-sectional studies**		

Hou et al., 2019	- 19–22 year old males - Rowers (*n* = 16) training for 1–2 h/day, 6 day/week, and sedentary controls (*n* = 16) provided resting blood samples	- Rowers: 24 h - Sedentary: ≥7 days	- Separation by differential ultracentrifugation - Particle size and concentration quantified by NTA - Western blot of positive EV markers CD81 and Tsg101 - Small EVs visualized with TEM	- No differences between groups	- miR-342-5p higher (↑1.8x) in Rowers compared to Sedentary when measured via qPCR
Nair et al., 2020	- >65 year old males - Trained (*n* = 5) or Sedentary (*n* = 5) stratified based activity history provided resting blood samples	48 h (personal communication)	- Separation by differential ultracentrifugation combined with commercial assay (ExoQuick) - Particle size and concentration quantified by NTA	- No differences between groups	- Small RNA-seq performed (Illumina MiSeq) with 7 miRNAs differentially expressed (> ± 2x and *P* < 0.05) in Trained vs. Sedentary - Higher: miR-206, miR-148a-3p, miR-486-5p, let7b-5p - Lower: miR-874-3p, miR-339-5p, miR-383-5p
Garai et al., 2021	- Sedentary humans aged 23 ± 2 year (*n* = 14; M/F, 2/12) compared to older males aged 62 ± 6 year (*n* = 11) identified as >25 year of training at least twice per week in aerobic and resistance exercise	At least 24 h	- Separation from pooled serum samples using Total Exosome Isolation reagent and traditional centrifugation - Particle size and concentration quantified by NTA, using unlabeled and labeled (CD63, CD81) preparations - Small EVs visualized with TEM	- No differences between groups	- 54 miRNA identified via NanoString multiplex array with 3 miRNAs differentially expressed in Trained vs. Sedentary (FDR < 0.05) - Decreased: miR-199a/b-3p (FC 0.40), miR-451a (FC 0.46), miR-23a-3p (FC 0.47)

*Several other studies have been performed that purported to investigate the effect of exercise training on the profile of circulating small EVs (Chaturvedi et al., 2015; Bei et al., 2017; Bertoldi et al., 2018; Barcellos et al., 2020; Xiang et al., 2020; Gao et al., 2021), but as referred to in the main text have been excluded because of their sampling of blood <24 h after cessation of the last exercise bout (see sections “‘Last Bout Effect’: The Importance of Sample Timing” and “Results From Rodent Studies”). Abbreviations: EPC, endothelial progenitor cells; FC, fold change; FDR, false discovery rate; NTA, nano-particle tracking analysis; and TEM, transmission electron microscopy.*

### Results From Rodent Studies

Nine studies have investigated the influence of exercise training on the small EV profile of rodents (Chaturvedi et al., 2015; Bei et al., 2017; Bertoldi et al., 2018; Ma et al., 2018; Hou et al., 2019; Barcellos et al., 2020; Castaño et al., 2020; Xiang et al., 2020; Gao et al., 2021). All of these studies were specifically interested in the miRNA cargo of small EVs, their differential expression and potential biological relevance, and employ an experimental design wherein animals subjected to aerobic exercise training are sampled after the cessation of training. Preparations of small EVs were then separated from plasma (Bei et al., 2017; Ma et al., 2018; Barcellos et al., 2020; Castaño et al., 2020; Xiang et al., 2020; Gao et al., 2021) or serum (Chaturvedi et al., 2015; Bertoldi et al., 2018; Hou et al., 2019), and compared to those of sedentary controls. Collectively, these studies have been inconsistent in their approaches toward separating and characterizing small EVs, have tended to measure only a limited number of specific miRNA as representative of small EV cargoes, and have reporting or methodological issues, which all combined, interferes with the inferential utility of these works.

For example, C57BL/6 mice subjected to 4 weeks of treadmill running at either a low (5 m/min) or moderate (10 m/min) speed, and sampled 24 h after the last exercise bout, had an increase in the resting abundance of both CD34^+^/VEGFR2^+^ small EVs and associated miR-126 cargo, in an intensity-dependent manner (Ma et al., 2018). These findings suggest that exercise training induces an increase in the resting concentration of some small EVs, namely those derived from endothelial progenitor cells based on their status as CD34^+^/VEGFR2^+^. However, in this study, the CD34^+^/VEGFR2^+^ small EVs were extracted from an initial separation of small EVs via differential ultracentrifugation using an additional procedure involving magnetic bead sorting. While a particle count assay (NTA) of these preparations estimated a ∼2 to 2.5-fold increase in particles in trained compared to sedentary groups, no difference was visually-apparent in the abundance of either CD34 or VEGFR2 markers in associated Western blots, and these markers were not quantified. As NTA is a non-specific method of small EV quantification, these results imply a disagreement between assays and call into question the inference of the increased resting concentration of small EVs in response to exercise training.

Additionally, Sprague-Dawley rats subjected to 4 weeks of swim exercise and sampled 24 h after the last exercise bout exhibited no change in NTA signal or the abundance of selected small EV markers (TSG101 and CD81) compared to controls, i.e., the basal concentration of small EVs was unchanged by training (Hou et al., 2019). Interestingly, however, 12 miRNA were observed to be differentially expressed (11 up-regulated, 1 down-regulated) in the small EV samples from the exercise-trained rats, suggesting that, even in the absence of a change in the resting concentration of small EVs, the cargo of small EVs may change with exercise training (Hou et al., 2019).

Four other studies have reported increases in signals indicative of particle enrichment and small EV marker abundance from small EV samples from exercise-trained rats (Bei et al., 2017; Barcellos et al., 2020) and mice (Chaturvedi et al., 2015; Gao et al., 2021). However, of these studies Barcellos et al. (2020) sampled 1 h after the last exercise bout, Gao et al. (2021) sampled immediately after the last exercise bout (personal communication), and Bei et al. (2017) sampled on average 6 to 12 h after the last exercise bout (personal communication), i.e., each study sampled in the immediate post-exercise period, when acute exercise-induced enrichment may still be present. We were unable to ascertain the time point of sampling from the work of Chaturvedi et al. (2015) or Xiang et al. (2020). Regardless, in training studies where sampling is in close proximity to the end of the last exercise bout, it is difficult, or sometimes impossible, to parse out whether results are indicative of the effect of acute exercise, residual bout effects, a true training effect, or a combination of the three.

Other studies have waited 24 h before taking post-training samples (Ma et al., 2018; Hou et al., 2019), but residual influences of an acute exercise bout may still be apparent within these and shorter time periods (section “‘Last Bout Effect’: The Importance of Sample Timing”). Indeed, in humans, acute exposure to downhill running and plyometric exercise produced decreases of miR-31b in preparations of small EVs for up to 24 h after exercise (Lovett et al., 2018). To our knowledge, only one other study has investigated the influence of exercise training on circulating small EV profiles in samples extracted >24 h after the last training bout (Castaño et al., 2020). Castano et al. subjected male C57BL/6 mice to 5 weeks of progressive high intensity treadmill running, and small EVs were isolated from plasma 48 h after the last training bout. No differences were observed in small EV abundance via an acetylcholinesterase assay, or subjective appraisal of Western blots of three small EV markers. Additionally, it should be noted, however, that acetylcholinesterase has been proven to not be a generic marker of EVs (Liao et al., 2019). Regardless, profiling of 378 miRNA transcripts by qPCR revealed seven differentially expressed miRNAs (6 miRNA with higher abundance, 1 miRNA with lower abundance) when comparing exercise-trained to sedentary groups (Castaño et al., 2020).

Collectively in these rodent studies, methodological limitations in terms of providing adequate indication of the presence of small EVs within samples separated from biofluids, and potential confounding by residual influences of the last exercise bout must be acknowledged, but some reports do indicate altered miRNA cargo within preparations of small EVs taken at rest after a period of exercise training (Hou et al., 2019; Castaño et al., 2020).

### Results From Human Studies

To our knowledge, there are currently four reports describing the influence of prior exercise training on preparations of circulating small EVs derived from resting humans (Hou et al., 2019; Nair et al., 2020; Estébanez et al., 2021; Garai et al., 2021; Table 2), three of which separated small EVs from plasma (Hou et al., 2019; Nair et al., 2020; Estébanez et al., 2021) and one which has separated small EVs from serum (Garai et al., 2021). The first study reported 1.8-fold greater abundance of miR-342-5p at rest in preparations of small EVs derived from young (19 to 22 years) male rowers with at least 1 year of training experience compared to sedentary controls (*n* = 16 in each group). However, small EVs were not formally characterized in this study, despite a small EV separation protocol being applied to plasma samples, and miR-342-5p abundance measured as the only target of interest. The next study compared resting miRNA profiles (via RNA sequencing) of small EV samples derived from endurance-trained and sedentary older (∼69 years) males, albeit with only *n* = 5 in each group (Nair et al., 2020). Seven differentially expressed miRNAs were identified between trained and untrained individuals, with 4 increased and 3 decreased in preparations of small EVs derived from the trained individuals. However, this study provided minimal information regarding the characterization of small EVs in samples by reporting only a single NTA result and making no comparisons between groups. Additionally, while the aerobic fitness of the participants was discordant (VO_2__max_ of 34.4 ± 1.1 and 21.7 ± 1.2 ml kg^–1^ min^–1^ in trained and untrained groups, respectively), and the trained participants clearly had superior fitness, the average VO_2__max_ for these individuals is still approximately half of what is commonly-reported for well-trained athletes of younger age (Jeukendrup et al., 2000; Jones et al., 2020). Therefore it is unclear whether a greater magnitude of fitness at younger age would create additional or alternative differences in the profile of resting small EVs between either the trained or sedentary group in the aforementioned study (Nair et al., 2020). The third study was in male and female older adults (∼73 years) that compared an 8 week resistance exercise training intervention (*n* = 28) to a sedentary control group (*n* = 10; Estébanez et al., 2021). However, the analysis was limited to the presence of small EVs via quantification of total exosome protein, identification of six marker proteins of small EVs via Western blot, and one proposed miRNA cargo of small EVs (miR-146a-5p; Estébanez et al., 2021). Of these, only the small EV marker CD63 exhibited a differential pattern between groups with the increase of ∼7% in the training groups being less than the ∼43% increase in the sedentary control group. Therefore, an attenuation of small EVs expressing CD63 may be a response to resistance exercise training, but overall, the data from this study are also limited in their coverage.

The final study consisted of two pilot experiments that investigated the potential influence of exercise training on the circulating profile of small EVs at rest (Garai et al., 2021). The first experiment consisted of healthy participants (*n* = 14; M/F, 2/12) performing a concurrent aerobic and resistance exercise training intervention 3 days per week for 6 months. Resting blood samples were taken at baseline and after the end of the training intervention, with each sample being reported as taken at least 24 h after any vigorous exercise. In this experiment, no difference was observed in the number of particles within separated preparations of small EVs via NTA before and after exercise training, although seven miRNA were differentially-expressed (all decreased) when analyzed using a NanoString array (Table 2). The second experiment reported in this study was a cross-sectional study in which resting profile of circulating small EVs from older men (aged 62 ± 6 years; *n* = 11) self-reporting > 25 years of exercise training [and identified as “trained” via their responses to the International Physical Activity Questionnaire (IPAQ; Craig et al., 2003)], was compared to the baseline sedentary samples of the cohort in the first experiment (Garai et al., 2021). Again no difference was observed in the resting abundance of small EVs between the older trained men and younger sedentary individuals via NTA. However, three miRNA were differentially-expressed (all decreased) when analyzed using a NanoString array (Table 2). Given that the older participants were defined as trained based on their response to the IPAQ rather than objective measures of exercise training history or physical fitness, arguably it is difficult to discern the extent to which these (minor) differences in miRNA were solely the result of divergent exercise training habits. Age and sex may also have been a factor given that the trained cohort in this study were on average ∼39 years older than the sedentary participants and were male as opposed to mostly females in the sedentary group (Garai et al., 2021). The resting profile of circulating small EVs is influenced by biological sex and chronological age (Noren Hooten, 2020) and therefore are likely to be major confounders in these data.

### Summary

Appraisal of existing studies across rodents and humans regarding the influence of exercise training on the abundance of small EVs and their proposed miRNA cargoes leads us to conclude that the characterization of small EVs within samples is generally inadequate by way of employing insufficient methodological approaches. Therefore, while some data indicate that the resting concentration of small EVs may change with exercise training, which has been commented by others (Brahmer et al., 2020; Nederveen et al., 2021), arguably the methods employed do not produce sufficient information to confidently state this to be the case.

Aside from the difficulty in accurately characterizing small EVs and their cargo, it is pertinent to also consider physiological and teleological aspects of the proposition that resting profiles would indeed be changed by exercise training. Circulating concentrations of small EV are a dynamic balance, such that the concentration measured in a resting sample is indicative of the processes of small EV release and small EV uptake, and yet small EVs may have a relatively short half-life of ∼7 min (Matsumoto et al., 2020). Thus, for enriched concentrations of small EVs to be observed in a resting sample distal to acute exercise, exercise training would have to induce augmented small EV release, attenuated small EV uptake, or both, at rest. To rigorously determine whether either or both occur would require determining which mechanisms govern systemic release and clearance of small EVs, and how exercise training either augments or attenuates these processes. Distinct from assessing small EV abundance, there is greater consistency across studies indicating that exercise training influences the miRNA profile of preparations of small EVs from resting samples. As exercise training can alter the transcriptomes (Pillon et al., 2019) and proteomes (Holloway et al., 2009; Ferreira et al., 2014; Padrão et al., 2016) of contractile tissues, a change in cargo profile is plausible independent of change in small EV abundance, because local changes to the biomolecular environment of a cell could alter the cargo that is released, even in the absence of an altered rate of EV release. However, it is important to reiterate that without adequate characterization of small EVs, the extent to which these differential miRNA profiles associate specifically to small EVs (even when small EV separations have been undertaken) is unknown. There remains the question as to why a persistent, as opposed to acute, change in resting profile of small EVs would occur with exercise training, and therefore, lastly we will consider the bioactivity of small EVs isolated from trained compared to untrained rodents and humans.

## Bioactivity of Small Extracellular Vesicles Obtained From Trained and Untrained Individuals

Consideration of the bioactivity and/or beneficial physiological effects of small EVs may provide insight into whether training-induced changes in the resting profile is a potential mediator of the benefits of exercise training, or perhaps methodological artifact. Six studies to our knowledge have performed *in vivo*/*ex vitro* experiments to investigate such effects (Bei et al., 2017; Ma et al., 2018; Hou et al., 2019; Castaño et al., 2020; Wang et al., 2020). However, we have not included discussion of two of these studies due to the post-training sample time point being <24 h after the last exercise training bout (Bei et al., 2017; Gao et al., 2021).

Of the remaining studies, two have investigated the bioactive effects of preparations of small EVs derived from exercise-trained mice exclusively in cell lines (Ma et al., 2018; Wang et al., 2020), and the other two have investigated potential *in vivo* effects through injection of small EVs derived from exercise-trained rodents into sedentary control animals (Bei et al., 2017; Castaño et al., 2020). In the first study (Ma et al., 2018), preparations containing CD34^+^/VEGFR2^+^ small EVs were co-incubated with cultured brain endothelial cells that were exposed to 18 h of 25 mM glucose and/or 6 h of hypoxia (1% O_2_, 5% CO_2_) as a model of endothelial cell injury. Co-culturing of cells with small EVs derived from exercise-trained or sedentary C57BL/6 mice produced reductions in cell apoptosis of ∼5, 10, and 20% for sedentary, low and moderate exercise-trained samples, respectively. Cell migration and tube formation was increased similarly in a condition-dependent manner. These effects were speculated to be mediated by miR-126, a transcript increased in preparations derived from the exercise-trained mice. In these cells, preparations of small EVs enhanced the expression of the angiogenic protein VEGF, while anti-miR-126 decreased VEGF expression and removed the described effects of small EV co-incubation (Ma et al., 2018). These results suggest that exercise training enhanced the abundance of specific transcripts within preparations of small EVs, which in turn are capable of enhancing the expression of local factors associated with some exercise adaptations, such as angiogenesis in this case.

Similar findings were reported in an *in vivo* model of cardiac injury induced in Sprague-Dawley rats (Hou et al., 2019). Sedentary rats received a direct intramyocardial injection of small EVs derived from exercise-trained rats 2 days prior to a surgically-induced myocardial infarct. Rats who had received injections of preparations of small EVs derived from exercise-trained counterparts demonstrated ∼40% lower infarct size 24 h post-surgery, and enhanced cardiac function through a lower reduction in ejection fraction and fractional shortening 4 weeks post-surgery, compared those injected with small EVs from sedentary controls. These results were similarly associated with the presence of a single miRNA in small EV samples (miR-342-5p), which was measured to reduce the abundance of some apoptotic signaling proteins (Caspase-9 and Jnk2), and potentiate proliferative Akt signaling, in cultured cardiomyocytes exposed to hypoxic damage (Hou et al., 2019). In follow-up work to Ma et al., Wang et al. again used preparations containing CD34^+^/VEGFR2^+^ small EVs derived from plasma from exercise-trained mice (60 min/d at 10 m/min, 5 day/week for 4 weeks). Co-incubation of these EV preparations with neuronal N2a cells subjected to hypoxia-reoxygenation injury resulted in protection against injury (increased cell viability, decreased apoptosis), and restored neurite length. These results coincided with greater secretion of brain-derived neurotrophic factor from these cells, and all effects were partially attenuated when inhibition of miR-126 or Akt-PI3K signaling was employed (Wang et al., 2020).

The work in each of these three studies should be noted as models that have potentiated recovery from injury in damaged cells or tissues (Ma et al., 2018; Hou et al., 2019; Wang et al., 2020) and therefore, these bioactivities do not necessarily translate into potentiating the function of healthy cells, or provide insight into mediating an exercise adaptation or training response in tissues. However, recent work by Castano et al. has attempted to address these questions. C57BL/6 mice were exposed to 4 weeks of high intensity interval training via treadmill running after which small EVs were separated from plasma collected 48 h after the last exercise training bout. These preparations, as well as preparations derived from sedentary control mice, were then injected intravenously daily for 4 weeks into separate groups of sedentary mice. At the end of the 4 week administration period, mice treated with exercise-trained small EVs demonstrated lower body mass (∼6%) and epidydimal fat mass, lower circulating triglycerides (∼15%), improved lipid tolerance, and improved insulin sensitivity and glucose tolerance (glucose AUC ∼35% lower), compared to the control mice (Castaño et al., 2020). Analysis of differentially-expressed miRNA between preparations of small EVs derived from exercise-trained or sedentary mice (discussed in section “Results From Rodent Studies” and Table 2) also yielded a thematic association with the regulation of the transcription factor forkhead box O1 (*FoxO1*), which in part regulates hepatic gluconeogenesis (Puigserver et al., 2003). Preparations of small EVs derived from the exercise-trained mice reduced hepatic *FoxO1* mRNA expression, as well as transcripts of several gluconeogenic proteins associated with *FoxO1* signaling. Thus, extended administration of small EV samples derived from exercise-trained mice was proposed to enhance glucose tolerance through small EV-mediated delivery of miRNAs that attenuated hepatic gluconeogenesis (Castaño et al., 2020).

**FIGURE 2:**
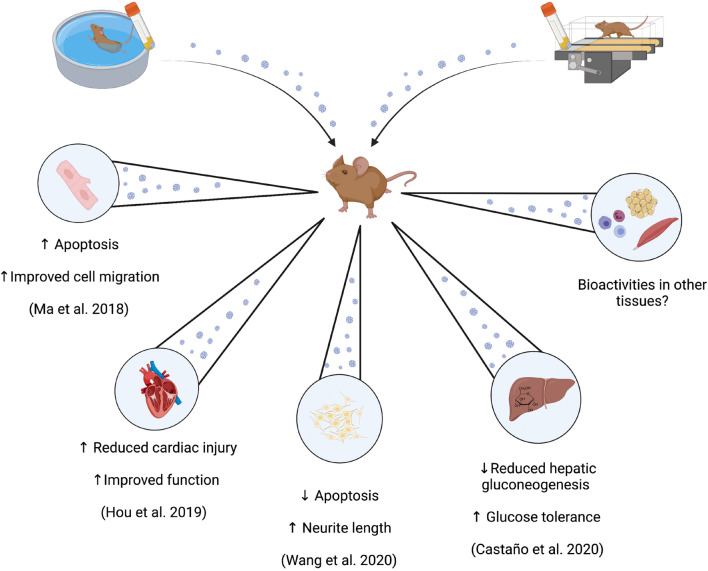
Potential bioactivities of circulating small EVs derived from exercise-trained rodents. Administration of circulating small EVs prepared from blood samples from exercise-trained rodents are identified to induce effects both *in vivo* and *ex vivo* that may be associated with health primarily as enhanced glucose tolerance (*in vivo)*, and enhanced recovery from ischemic injury (*in vivo* and *in vitro*).

In summary, small EVs derived from resting blood samples of exercise-trained rodents appear to exert bioactive, and potentially beneficial, effects in several tissues compared to sedentary counterparts (Figure 2). These results suggest that exercise-trained small EV samples may offer therapeutic potential in specific use cases, or may play a role in exercise adaptation, and/or homeostatic maintenance. However, more research is required to determine aspects such as the consistency of specific metabolic effects, the type and time course of exercise training required to induce reliable changes in resting small EV profiles in humans, the duration for which these changes are sustained after training cessation, and whether it is the entire small EV sample or specific cargo within those samples that are the strongest determinants of observed bioactivity.

## Concluding Remarks

The purpose of this review was to appraise the currently-available evidence regarding exercise-induced changes in the circulating profile of small EVs against an established biological paradigm of the response of their proposed cargo, i.e., exercise factors. On the whole, presently the small EV profile may indeed be changed both in response to acute exercise (via both altered small EV abundance and cargo), and at rest (via altered cargo profiles) in the exercise-trained state. However, working with small EVs is difficult and requires rigorous approaches to both separation and identification, which requires employing multiple complex methodologies. In this regard, the results of many studies are somewhat limited, as essential aspects of small EV characterization and/or quantification are often absent. This, in turn, has implications for the extent to which the role of EVs in exercise metabolism and adaptation can be understood.

Many pertinent questions remain, such as the proportional contribution of different cell types to the circulating small EV pool both at rest and with exercise, and by which mechanisms in cells a shift may occur in the cargoes that are packaged and released from small EVs during exercise or after training. Finally, investigation of the time course for which the circulating small EV profile at rest may change in response to exercise will inform whether training-induced changes in exercise factors are primarily residual artifacts, or an important component of the mechanistic basis for exercise adaptation. At present there are promising preliminary data that small EV preparations from exercise-trained samples do exert relevant bioactivity that may be important for the beneficial effects of exercise in organs beyond skeletal muscle.

## Author Contributions

ID and BE conceptualized the review. ID wrote and developed the initial drafts of the manuscript with feedback from BE. BE and ID wrote and developed advanced drafts of the manuscript with feedback from LO’D. BE developed and formatted the table and ID illustrated all figures, which were edited based on feedback from BE and LO’D. All authors contributed to the article and approved the submitted version.

## Conflict of Interest

The authors declare that the research was conducted in the absence of any commercial or financial relationships that could be construed as a potential conflict of interest.

## Publisher’s Note

All claims expressed in this article are solely those of the authors and do not necessarily represent those of their affiliated organizations, or those of the publisher, the editors and the reviewers. Any product that may be evaluated in this article, or claim that may be made by its manufacturer, is not guaranteed or endorsed by the publisher.
